# Feedback Control Architecture and the Bacterial Chemotaxis Network

**DOI:** 10.1371/journal.pcbi.1001130

**Published:** 2011-05-05

**Authors:** Abdullah Hamadeh, Mark A. J. Roberts, Elias August, Patrick E. McSharry, Philip K. Maini, Judith P. Armitage, Antonis Papachristodoulou

**Affiliations:** 1 Department of Engineering Science, University of Oxford, Oxford, United Kingdom; 2 Oxford Centre for Integrative Systems Biology, Department of Biochemistry, Oxford, United Kingdom; 3 Department of Biochemistry, University of Oxford, Oxford, United Kingdom; 4 Mathematical Institute, University of Oxford, Oxford, United Kingdom; 5 Centre for Mathematical Biology, Mathematical Institute, University of Oxford, Oxford, United Kingdom; North Carolina State University, United States of America

## Abstract

Bacteria move towards favourable and away from toxic environments by changing their swimming pattern. This response is regulated by the chemotaxis signalling pathway, which has an important feature: it uses feedback to ‘reset’ (adapt) the bacterial sensing ability, which allows the bacteria to sense a range of background environmental changes. The role of this feedback has been studied extensively in the simple chemotaxis pathway of *Escherichia coli*. However it has been recently found that the majority of bacteria have multiple chemotaxis homologues of the *E. coli* proteins, resulting in more complex pathways. In this paper we investigate the configuration and role of feedback in *Rhodobacter sphaeroides*, a bacterium containing multiple homologues of the chemotaxis proteins found in *E. coli*. Multiple proteins could produce different possible feedback configurations, each having different chemotactic performance qualities and levels of robustness to variations and uncertainties in biological parameters and to intracellular noise. We develop four models corresponding to different feedback configurations. Using a series of carefully designed experiments we discriminate between these models and invalidate three of them. When these models are examined in terms of robustness to noise and parametric uncertainties, we find that the non-invalidated model is superior to the others. Moreover, it has a ‘cascade control’ feedback architecture which is used extensively in engineering to improve system performance, including robustness. Given that the majority of bacteria are known to have multiple chemotaxis pathways, in this paper we show that some feedback architectures allow them to have better performance than others. In particular, cascade control may be an important feature in achieving robust functionality in more complex signalling pathways and in improving their performance.

## Author Summary

Bacteria move towards favourable environments by changing their swimming pattern. An important feature of this response, which is called *bacterial chemotaxis*, is that their sensing ability remains independent of the background environment in which they find themselves. This feature has been studied extensively in the bacterium *E. coli*, which has a simple chemotaxis decision mechanism. However, it has been recently found that most bacteria could potentially have a much more complicated decision mechanism for this response. In this paper, we look at the chemotaxis behaviour of one such bacterium, *R. sphaeroides*. We develop mathematical models of possible decision mechanisms and undertake an experimental procedure to investigate their validity. We find that only one of four such models can explain the chemotaxis response in *R. sphaeroides*. Compared to the other models, this model corresponds to a decision mechanism that provides the bacterium with improved swimming performance over the others. Moreover, this decision mechanism has been used extensively to improve performance in several engineering systems. We suggest that this mechanism may play an important role in improving chemotactic performance in other bacteria and in other signalling pathways.

## Introduction

Living organisms respond to changes in their internal and external environment in order to survive. The sensing, signalling and response mechanisms often consist of complicated pathways the dynamical behaviour of which is often difficult to understand without mathematical models [1]. Considering the structure and dynamics of these signalling pathways as integrated dynamical systems can help us understand how the pathway architecture and parameter values result in the performance and robustness in the response dynamics [2].

One extensively studied sensory pathway is bacterial chemotaxis. This pathway controls changes in bacterial motion in response to environmental stimuli, biasing movement towards regions of higher concentration of beneficial or lower concentration of toxic chemicals. The chemotaxis signalling pathway in the bacterium *Escherichia coli* is a simple network with one feedback loop [3] which has been extensively studied and used as a paradigm for the mechanism of chemotaxis signalling networks [4]. In *E. coli*, chemical ligands bind to methyl-accepting chemotaxis protein (MCP) receptors that span the cell membrane and alter the activity of a cytoplasmic histidine kinase called CheA. When attractant ligands stimulate the chemotaxis pathway by binding to MCP, there is a decrease in the autophosphorylation rate of CheA; conversely, repellent binding or lack of attractant binding increase CheA autophosphorylation activity. CheA, when phosphorylated, can transfer the phosphoryl group to two possible response regulators: CheY and CheB. CheY-P (where ‘-P’ denotes phosphorylation) interacts with FliM in the multiple *E. coli* flagellar motors resulting in a change in the direction of rotation of the motor. At the same time, a negative feedback loop allows the system to sense temporal gradients and react to a wide ligand concentration range: the MCP receptors, which are constantly methylated by the action of a methyltransferase CheR, are de-methylated by CheB-P. This negative feedback loop restores the CheA autophosphorylation rate and the flagellar activity to the pre-stimulus equilibrium state [5], [6].

Describing this pathway mathematically as a dynamical system can be facilitated by using tools from control theory. For example, it has been shown that the adaptation mechanism in the *E. coli* model [7], [8] is a particular example of integral control, a feedback system design principle used in control engineering to ensure the elimination of offset errors between a system's desired and actual signals, irrespective of the levels of other signals [9].

Many species have chemotaxis pathways that are much more complicated than that of *E. coli* [10], [11], either containing chemotaxis proteins not found in *E. coli*, e.g. in the case of *Bacillus subtilis* [12]; or containing multiple homologues of the proteins found in the *E. coli* pathway, as in the case of *Rhodobacter sphaeroides* [11], [13]. Furthermore, in *R. sphaeroides* there are two receptor clusters containing sensory proteins which localize to different parts of the cell, one located at the cell pole and the other in the cytoplasm [14]. Although the purpose of the two clusters is unclear, *in vitro* phosphotransfer experiments [15], [16] show that the CheA homologues located at the two clusters can phosphotransfer to different CheY and CheB homologues: at the cell pole CheA_2_-P phosphotransfers to CheY_3_, CheY_4_, CheY_6_, CheB_1_ and CheB_2_, while at the cytoplasm CheA_3_A_4_-P phosphotransfers to CheY_6_ and CheB_2_. The two methylesterase proteins, CheB_1_ and CheB_2_, which are homologues of CheB in *E. coli*, are responsible for the adaptation mechanism in *R. sphaeroides* [13], [17]. Past localization studies have shown that CheB_1_ and CheB_2_ are found diffuse throughout the cytoplasm [14]. This is different to *E. coli* where the CheB protein is localized at the cell pole, and could potentially mean that the two proteins de-methylate either receptor cluster [14].

As a system featuring an adaptation mechanism similar to that in *E. coli*, but with multiple homologues of the *E. coli* chemotaxis proteins, it is useful to examine the *R. sphaeroides* chemotaxis pathway from a control engineering perspective. In this way, we can suggest structures for the *R. sphaeroides* chemotaxis pathway that integrate the control mechanisms thought to be responsible for adaptation in *E. coli* along with the possible feedback architectures that arise from the dual sensory modules present in *R. sphaeroides*. The relative evolutionary advantages of the different architectures can then be compared from both control engineering and biological points of view. The fact that there are two endogenous ‘measurements’ available to the feedback mechanism (CheB_1_-P and CheB_2_-P) which can be used to regulate two signals (CheA_2_ and CheA_3_A_4_) makes the whole chemotaxis feedback pathway a multi-input, multi-output control system (as opposed to possessing only one CheB and one CheA as in the *E. coli* models [7], [18]). This introduces extra degrees of freedom in the feedback control mechanism of the system and, thus, the potential for better regulation.

However, the different conceivable connectivity configurations between the two CheB-P proteins and the two receptor clusters actually correspond to different feedback control architectures, each with different properties. Some of these configurations, as will be demonstrated, could allow the bacterium to integrate information from both internal and external sources and to function more efficiently, e.g., by varying how strongly it reacts to external attractants depending on its internal state. At the same time, the additional receptor cluster not found in *E. coli* has the potential of introducing extra sources of performance degradation such as noise (both intrinsic and extrinsic) and variations in quantities internal to the cell such as protein copy numbers and phosphorylation rates: the feedback signalling pathway may be required to remedy this, and in this regard, some of these feedback architectures perform better than others.

**Figure 1:**
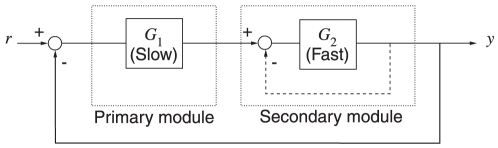
A cascade control system. The subsystem 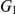 is slow relative to 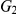. Cascade control involves placing a negative feedback loop (dashed line) around the fast secondary module. This scheme helps reduce the sensitivity of the system's output to uncertainties in the subsystems 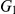 and 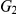.

One of the different pathway configurations that is possible in this system has similarities to a feedback architecture commonly found in engineering control systems termed *cascade control* [19], which is usually employed when the process to be controlled can be split into a slow ‘primary’ sub-process (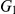 in Figure 1) and a faster, secondary sub-process (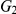 in Figure 1). Without the internal feedback shown dashed in Figure 1 the primary module maintains a set-point for the secondary module to follow and the output of the secondary module is fed back to the primary. A cascade control design places an additional feedback loop around the fast secondary process (shown dashed). This has been known to improve system performance in several ways: it reduces the sensitivity of the output of the secondary module to changes in the parameters (thus improving robustness), it attenuates the effects of disturbance signals, it makes the step response of the control system to inputs and disturbances less oscillatory and, since the secondary process is relatively fast, the effects of unwanted disturbances are corrected before they affect the system output. Including this additional internal feedback also allows the control system designer more flexibility in increasing the feedback gain to achieve higher bandwidth and faster system responses without losing stability. In fact, cascade control is employed as a design principle in several engineering systems such as aircraft pitch control and industrial heat exchangers (see Text S1 for further details).

**Figure 2:**
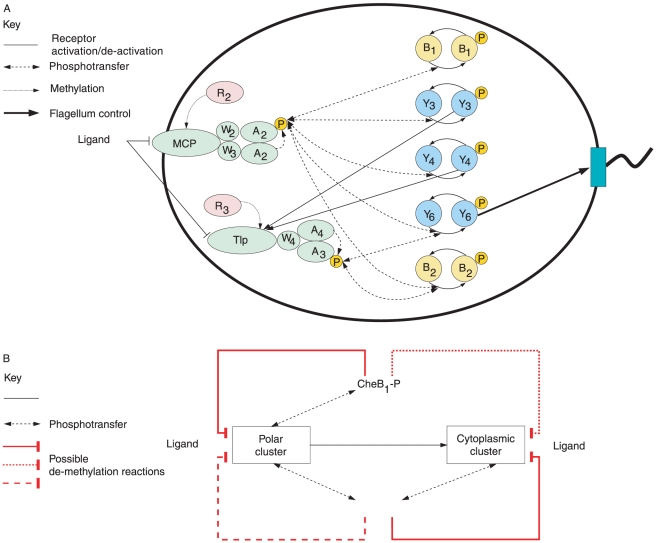
Chemotaxis in *R. sphaeroides*. (A) The chemotaxis pathway in *R. sphaeroides* as currently understood, including the forward chemotaxis pathway previously proposed [20]. MCP: transmembrane methyl accepting chemotaxis protein, Tlp: cytoplasmic methyl accepting chemotaxis protein, A: CheA histidine protein kinase, W: CheW a linker protein between receptors and CheA, Y: the response regulator CheY, B: the response regulator CheB, R: the methyltransferase CheR. P indicates a phosphoryl group. The number in subscript denotes one of the multiple homologues in *R. sphaeroides*. The flagella motor is shown at the right of the figure. (B) The possible de-methylation feedback structures for the phosphorylated proteins CheB_1_-P and CheB_2_-P in *R. sphaeroides*. Each possible connection is denoted by a (red) thick solid, dashed or dotted line. Possible models involve combinations of these four lines. Interactions from the phosphotransfer network are shown in (black) thin dashed arrows, receptor activation/de-activation is denoted by (black) thin solid lines.

In our previous work [20], we used a model invalidation technique to arrive at a possible pathway architecture that allows the *R. sphaeroides* chemotaxis system to convey, via a signalling cascade, sensed changes in ligand concentration outside the cell to the flagellar motor. In that model, proteins CheY_3_-P and CheY_4_-P act together to promote autophosphorylation of CheA_3_A_4_ (schematically illustrated in Figure 2(A)) whilst CheY_6_-P binds with the FliM rotor switch to increase the frequency of motor switching (and hence reduce the motor rotation frequency). This stimulation of CheA_3_A_4_ need not be a direct interaction [20].

In this paper, we assume that the chemotaxis pathway has the same forward signalling pathway of [20] and then suggest four plausible interconnection structures for the feedback pathway between the two CheB-P proteins and the two receptor clusters. Following this, we present the results of experiments that are used to invalidate all but one of these structures. We then discuss the results of *in silico* experiments that highlight the differences in chemotactic performance between the different models with particular focus on the robustness of chemotaxis to parametric variations in the chemotaxis pathway and noise [21], [22]. Using analytical techniques from control theory, we demonstrate that the model not invalidated by our experiments is structurally similar to the cascade control architecture, and we use the structural properties of this interconnection, which are commonly used to reduce the effects of uncertainty and disturbances in various engineering applications, to explain the robustness features of the suggested model.

## Results

### Chemotaxis model creation

**Figure 3:**
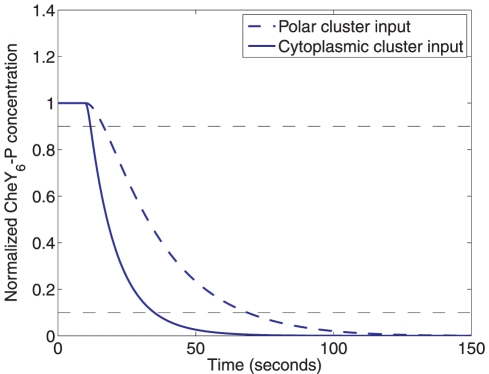
The speed of response of each cluster to input signals. The response of the normalized CheY_6_-P concentration to a step decrease, at time 10 seconds, in the number of active receptors at the polar cluster (from 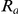 = 1 µM to 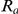 = 0 µM, dashed) and at the cytoplasmic cluster (from 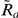 = 1 µM to 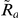 = 0 µM, solid). Such a decrease in active receptors can be due to a step increase in sensed ligand. A step decrease in active polar cluster receptors results in a slower fall in the normalized CheY_6_-P concentration (90%-10% fall time: 50.57 sec) than would an identical change in the number of active cytoplasmic cluster receptors (90%-10% fall time: 21.98 sec).

Given the structure of the forward path of the chemotaxis pathway from [20], illustrated in Figure 2(A), and given the rates previously measured in [15], [16] for the phosphotransfer reactions also shown in Figure 2(A), we constructed a generic ordinary differential equation model of the *R. sphaeroides* chemotaxis pathway, detailed in Materials and Methods. With this forward signalling pathway, the model makes the following assumptions:

- Polar and cytoplasmic cluster receptors are either methylated or un-methylated.
- Only a subset of methylated receptors is active, as in [23].
- CheR_2_/CheR_3_ act to methylate inactive receptors whilst proteins CheB_1_-P/CheB_2_-P de-methylate active polar and cytoplasmic receptors with unknown connectivity, as in [23].
- A sensed increase in ligand concentration causes a reduction in the number of active receptors.
- Active polar and cytoplasmic receptors promote the auto-phosphorylation of CheA_2_ and CheA_3_A_4_ respectively.
- CheY_3_-P and CheY_4_-P act together to promote autophosphorylation of CheA_3_A_4_ (Figure 3) whilst CheY_6_-P binds the FliM rotor switch to increase the frequency of motor switching.
- Through the phosphotransfer network, a decrease in the number of active receptors due to a sensed increase in ligand concentration results in a subsequent decrease in the amount of CheY_3_-P, CheY_4_-P, CheY_6_-P, CheB_1_-P and CheB_2_-P.

One effect of a sensed increase in ligand concentration is a decrease in the flagellar switching frequency due to decreased amounts of CheY_6_-P binding with FliM. Figure 3 shows the result of a simulation of the signalling pathway that demonstrates the fall in the concentration of CheY_6_-P in response to a step decrease in the number of active receptors at the polar or at the cytoplasmic clusters. The reaction rates of the phosphotransfer network are such that a change in the number of active receptors at the cytoplasmic cluster causes a faster fall in CheY_6_-P concentration than does a similar change in the number of active receptors at the polar cluster.

Qualitatively, the adaptation mechanism in the generic ODE model presented in Materials and Methods functions as follows: CheB_1_-P and CheB_2_-P are assumed to de-methylate active receptors, and the phosphotransfer network responds to a sensed increase in ligand concentration by reducing the concentration of CheB_1_-P, CheB_2_-P, CheY_3_-P, CheY_4_-P and CheY_6_-P. This results in a reduction in the de-methylation rate of active receptors in the two receptor clusters, and also results in a decrease in the flagellar stopping frequency (which corresponds to an increase in the flagellar rotation rate). The constant methylation of inactive receptors by CheR_2_ and CheR_3_ then causes the number of methylated receptors, and, it is assumed, of active receptors, to increase. Thus, the number of active receptors is eventually restored to its pre-stimulus equilibrium level. In turn, the phosphotransfer network then restores the amount of CheY_6_-P, and hence the flagellar switching frequency, back to its original level. According to the model of the forward signalling pathway, the proteins CheB_1_-P and CheB_2_-P therefore act as feedback signals that restore the chemotaxis pathway to its original state. However, the exact connectivity between CheB_1_-P/CheB_2_-P and the two receptor clusters is unknown.

To determine the most likely interconnection structure and to provide a rationale of how such a structure may be advantageous in terms of chemotactic performance, we created four variants of the generic ODE model with the forward pathway, each having a different interconnection structure between the proteins CheB_1_-P/CheB_2_-P and the two receptor clusters (Figure 2(B)). All models were able to produce wild type response data and behaved as expected for the response data generated with gene deletions available at the time. The unknown parameters in the models (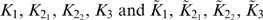) were fitted to wild type data for each model. The significance of these parameters is as follows:

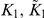
 : Along with the sensed ligand concentration, these parameters determine the proportion of methylated receptors that are active at the polar and cytoplasmic clusters respectively.

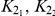
: These parameters determine the strength of the CheB_1_-P, CheB_2_-P feedbacks to the polar cluster respectively.

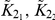
: These parameters determine the strength of the CheB_1_-P, CheB_2_-P feedbacks to the cytoplasmic cluster respectively.

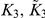
 : These parameters represent the activity of CheR_2_/CheR_3_ respectively.

For notational convenience, it is useful to group the CheB_1_-P/CheB_2_-P feedback gains 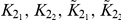 into a feedback matrix 
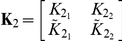
. The four CheB_1_-P and CheB_2_-P feedback connectivities (and their associated 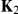) for which models were constructed are as follows:

- CheB_1_-P regulates the methylation state of the polar receptor cluster and CheB_2_-P of the cytoplasmic cluster only (shown in solid de-methylation reactions in Figure 2 (B)): 
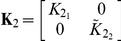
_._
- CheB_1_-P regulates the methylation state of both the polar cluster and the cytoplasmic cluster while CheB_2_-P de-methylates only cytoplasmic cluster receptors (solid de-methylation reactions and the dotted de-methylation reaction in Figure 2 (B)): 
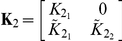
_._
- CheB_1_-P and CheB_2_-P both regulate the methylation state of the polar receptor cluster and CheB_2_-P of the cytoplasmic receptor cluster only (solid de-methylation reactions and the dashed de-methylation reaction in Figure 2 (B)): 
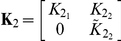
_._
- CheB_1_-P and CheB_2_-P both regulate the methylation state of both receptor clusters (solid de-methylation reactions, the dashed de-methylation reaction and the dotted de-methylation reaction in Figure 2 (B)): 
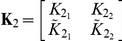
_._

**Figure 4:**
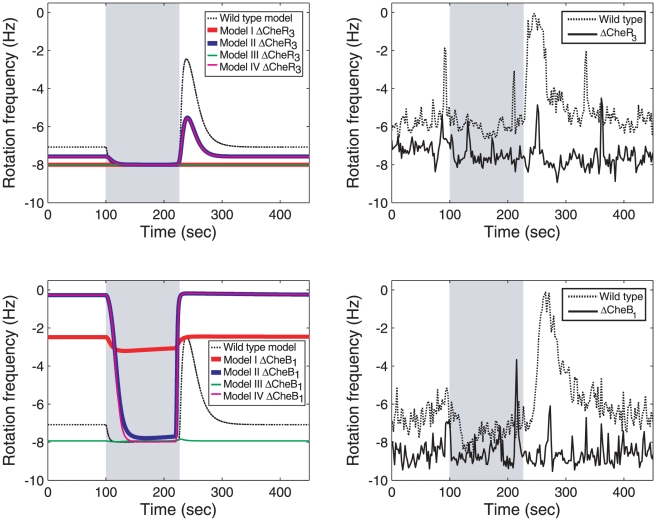
Model invalidation. Top left: Simulations of the wild type Models I–IV and with CheR_3_ deleted in response to 100 µM of ligand added at 100 seconds and removed at 220 seconds. Top right: Average responses of wild type cells and CheR_3_ deletion cells in a tethered cell assay with 100 µM of propionate added at 100 seconds and removed at 220 seconds. Bottom left: Simulations of the wild type Models I–IV (dashed line) and with CheB_1_ deleted in response to 100 µM of ligand added at 100 seconds and removed at 220 seconds. Bottom right: Average responses of wild type cells and CheB_1_ deletion cells in a tethered cell assay with 100 µM of propionate added at 100 seconds and removed at 220 seconds. Cells rotate counter clockwise hence negative Hz values are observed. Ligand addition is marked by grey shading.

After constructing these four models, we carried out experiments to differentiate between them, by finding the optimal initial conditions of the cells in the assay so as to maximize the difference between the outputs of the different models [20], [24]. The conditions searched were limited to what could be implemented experimentally and included deletions, over-expression of proteins and combinations of these. To confirm these conditions allow for invalidation, simulations were run of the four models I–IV testing the possible initial conditions and inputs. The simulations showed that the initial conditions that allow for the best model invalidation were the deletion of CheR_3_ and, in a separate experiment, the deletion of CheB_1_ (Figure 4).

**Figure 5:**
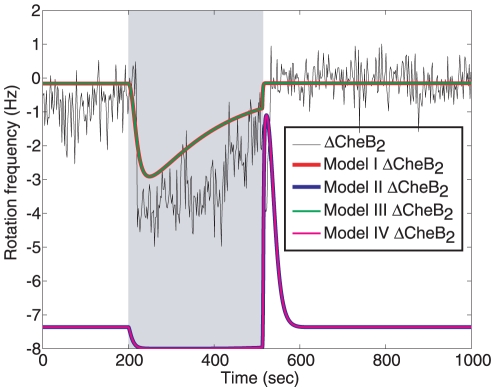
Deletion of CheB_2_. Average responses of CheB_2_ deletion cells in a tethered cell assay with 100 µM of propionate added at 200 seconds and removed at 512 seconds. Solid lines: simulations of the Models I–IV with CheB_2_ deleted in response to 100 µM of ligand added at 200 seconds and removed at 512 seconds. Cells rotate counter clockwise hence negative Hz values are observed. Ligand addition is marked by grey shading.

The experiments were then implemented in *R. sphaeroides*, subjecting a population of cells to a step increase in ligand concentration (propionate) and then measuring the resulting flagellar activity through a tethered cell assay (Figure 4). Experimentally the deletion of either CheB_1_ or CheR_3_ resulted in cells with a rotation frequency of −8 Hz that showed no noticeable response to the addition or removal of ligand. In the simulations, only Models I and III displayed this behaviour upon deletion of CheR_3_ (Figure 4, top row) and only Model III displayed this behaviour upon deletion of CheB_1_ (Figure 4, bottom row). Models I, II and IV were thus invalidated and only Model III was able to replicate the experimental data. As a test of this model invalidation, a further experiment wherein CheB_2_ was deleted was performed. The result of this experiment and the outputs of the four models under the CheB_2_ deletion (overlaid) are shown in Figure 5. Models I and III were once again able to replicate the deletion data whilst Models II and IV produced outputs that differed from the experimental outcome.

### Dynamic properties of chemotaxis models

The experiments described above demonstrated that the proposed Models I, II and IV are invalid, being unable to explain experimental data. To compare the four models further, *in silico* experiments were performed on the data-fitted Models I–IV that compared how the different feedback configurations affect chemotactic performance in terms of the sensitivity of the flagellar stopping frequency in response to variations in the values of the models' biochemical parameters and in response to noise. Following these results, we use linear models with structures that represent the different connectivities of Models I–IV to analyze these structures' relative sensitivities to parametric variations and noise.

### Chemotactic performance

**Figure 6:**
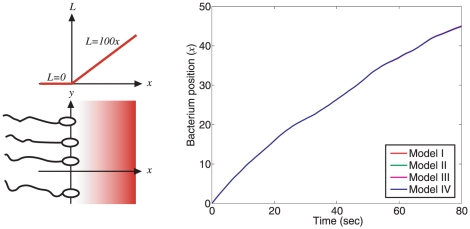
Comparison of chemotactic performance. The four chemotaxis models are simulated in a two-dimensional environment, wherein the chemoattractant concentration *L* has a ramp profile that varies along the *x*-direction only, such that *L* = 100*x* for *x*>0 and *L* = 0 otherwise (left). The simulation output (right) shows the relative average distance travelled up the attractant gradient by ten cells for each of the chemotaxis models.

The performance of the different chemotaxis models was compared by simulating the efficiency of each model in ascending an attractant gradient, as illustrated in Figure 6 (left). For each chemotaxis model, Figure 6 shows the average distance travelled up the attractant gradient by ten bacteria during a simulation lasting 80 seconds. As shown in Figure 6 (right), the chemotactic performances of the different models according to this measure were nearly identical (see Materials and Methods for more details).

### Response to noisy ligand variations

**Figure 7:**
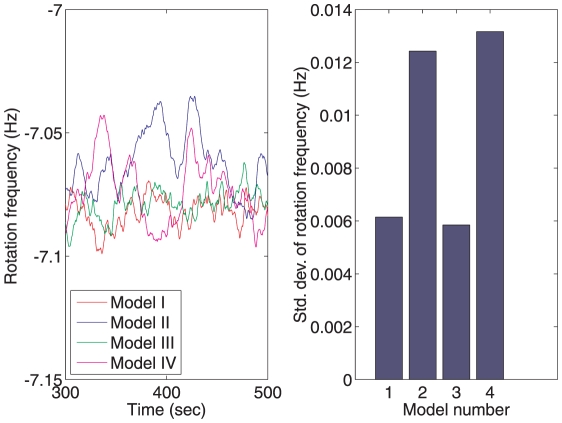
Response to external ligand variations. Standard deviations of the flagellar rotation frequencies for each of the four chemotaxis models in response to a noisy ligand input sensed at the polar cluster given by *L* = max(0,1+*η*) (where *η* is a white noise signal with a zero-mean, unity variance Gaussian distribution).

The bacterium's environment is typically composed of regions of high and low chemoattractant or chemorepellant concentrations. Additionally, the bacterium will sense small, fast fluctuations in the detected level of ligand due to molecular noise. To test how sensitive the chemotaxis Models I–IV are to such ligand fluctuations, an *in silico* experiment was performed on each model in which the ligand concentration sensed by the polar cluster, *L*, was modelled as the noisy signal *L* = max(0,1+*η*), where *η* is a white noise signal with a zero-mean, unit variance Gaussian distribution. The resulting rotation frequencies were then recorded and are shown in Figure 7. As can be seen in Figure 7, ligand level fluctuations sensed at the polar cluster of receptors resulted in larger variance of the rotation frequency in Models I, II and IV than in Model III.

**Figure 8:**
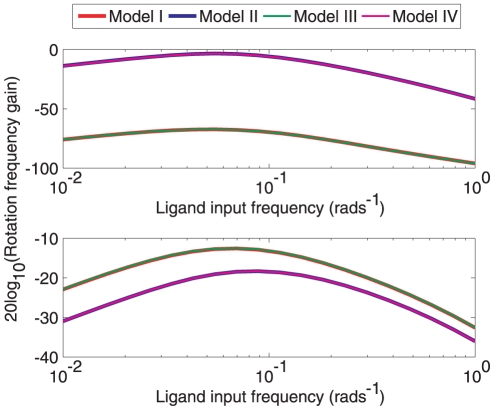
Input-output gains of the two sensing clusters. Frequency response magnitude plots showing the response of the different models to sinusoidally-varying ligand concentrations modelling noisy ligand input signals. Top: Constant ligand to cytoplasmic cluster and variable ligand to polar cluster (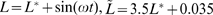, where 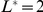). Bottom: Constant ligand to polar cluster, sinusoidal to cytoplasmic cluster (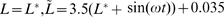, where 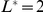).

The sensitivity of the chemotaxis Models I–IV to ligand inputs was then tested in two *in silico* experiments which were performed on each model and in which the flagellar rotation frequency was recorded in response to sinusoidal variations in the ligand signals 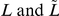 (the latter of which corresponds to ligand inputs acting on the cytoplasmic cluster). As can be seen in Figure 8, ligand level fluctuations sensed at the polar cluster of receptors resulted in larger changes in the rotation frequency in Models II and IV than in I and III. When the ligand concentration variations were sensed at the cytoplasmic cluster the result was a greater variation in the rotation frequency in Models I and III than in the other two models. Once more, these simulations suggest that CheB_1_-P de-methylating the cytoplasmic cluster differentiates the performance of Models II and IV from Models I and III.

### Parametric sensitivity analysis of the chemotaxis models

**Figure 9:**
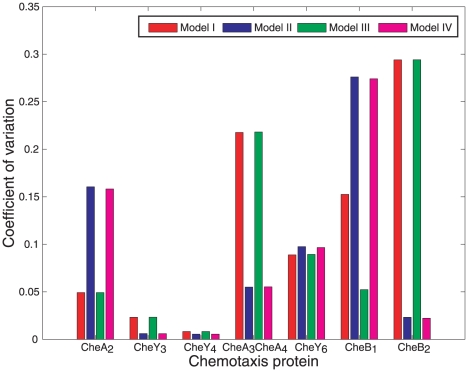
Parametric sensitivity analysis. Relative sensitivities of the rotation frequency outputs of the different chemotaxis models to changes in the chemotaxis protein copy numbers.

To investigate the sensitivity of the models to parameter variations, we performed an *in silico* experiment in which, for each of the different chemotaxis models, the variation of the steady-state of the chemotaxis system was measured under randomly chosen values of the copy numbers of chemotaxis proteins (see Materials and Methods). For each chemotaxis protein, the resulting coefficient of variation of the steady-state is shown in Figure 9. Once more, there was a similarity in the sensitivity of each model to these parametric variations between Models I and III and between Models II and IV, with the latter pair showing slightly higher sensitivity to copy numbers of the chemotaxis protein CheY_6_ among others. In addition, Model III showed considerably lower sensitivity with respect to CheB_1_ copy numbers than the other models.

### Linear model analysis

**Figure 10:**
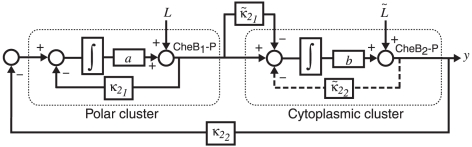
Comparison with engineering systems. Block diagram representation of a linear system structurally similar to the *R. sphaeroides* chemotaxis pathway. In this system, gain 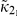 corresponds to 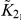 in the chemotaxis model, 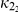 to 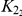, 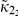 to 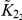 and 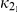 to 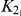. Levels of CheB_1_-P and CheB_2_-P exhibit exact adaptation to step changes in ligand concentration 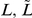. We assume 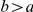, mirroring the faster dynamics of the cytoplasmic cluster relative to the polar cluster.

Further insight to the differences in performance between the models can be obtained by analyzing the interconnection structure of these models using control theory. In particular, the way in which such feedback arrangements can affect the performance of control systems like the *R. sphaeroides* chemotaxis pathway can be studied by comparing the behaviour of different linear systems that are structurally similar to Models I–IV. The block diagram in Figure 10 depicts a system composed of two modules representing the polar and cytoplasmic clusters. The CheB_1_-P/CheB_2_-P outputs of the two modules exhibit exact adaptation through integral control in response to step changes in the input ligand concentration level, as in *E. coli* [8]. Depending on the values of feedback gains 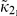 and 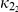 (which correspond to 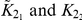 respectively in the chemotaxis models described above), the system can represent one of the four chemotaxis models:

Model I: 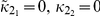

Model II: 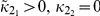

Model III: 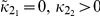

Model IV: 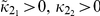.

The gains 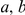 in Figure 10 are such that 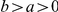, representing the fact that the cytoplasmic receptor cluster can, as a result of the measured reaction rates, relay a sensed ligand input signal to the flagellar motor faster than the polar receptors cluster (see Figure 3). For the examples we shall consider we set 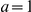 and 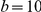. Gains 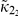 and 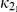 correspond to 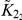 and 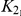 in the chemotaxis model respectively. The frequency domain transfer function of the system in Figure 10 from the ligand inputs 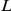 and 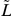 to the output 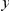 is then
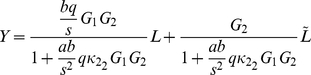
(1)where 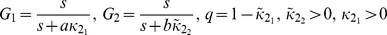. This function is a frequency-domain map from signals 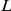 and 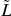 to the output *Y*, which corresponds to the flagellar rotation frequency. In the following, we shall use this frequency domain representation of the chemotaxis system to demonstrate how the feedback of linear systems with structures similar to the chemotaxis Models I–IV affects system performance.

**Figure 11:**
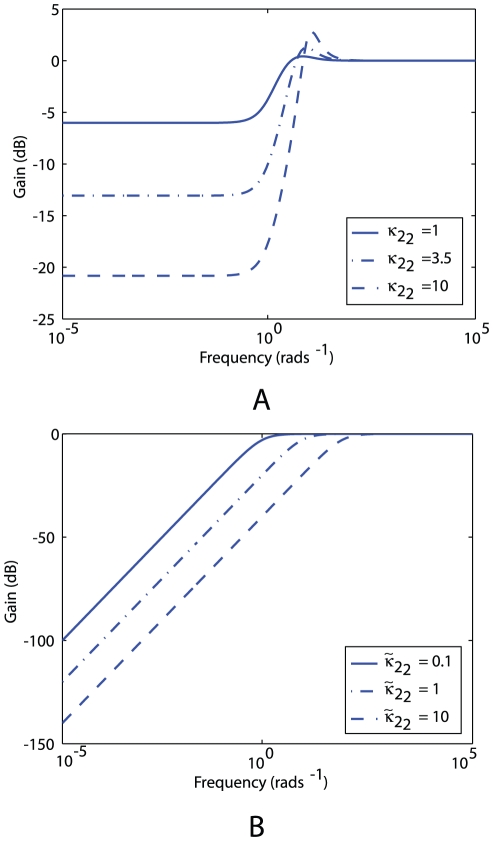
Variation of linear system sensitivity under different feedback strengths as a function of frequency. (A) Bode magnitude plots of the sensitivity function of system (1) with 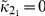 and different values of gain 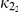, which corresponds to the feedback strength of CheB_2_-P de-methylating active polar cluster receptors. With these gains the system is structurally similar to Model III. (B) Sensitivity function of the block corresponding to the cytoplasmic cluster in the linear model (1), for different values of feedback gain 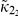, which corresponds to the feedback strength of CheB_2_-P de-methylating active cytoplasmic cluster receptors. The frequency domain sensitivity function is 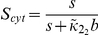 (see Text S1).

The Bode magnitude diagrams (Materials and Methods) in Figure 11(A) illustrate the effect of increasing 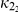 in reducing the sensitivity function of the system (1) over most excitation frequencies (see the Discussion and Text S1 for a brief introduction to sensitivity functions). At the same time, Figure 11(B) shows that strengthening the feedback 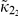, which corresponds to increasing the de-methylation of the cytoplasmic cluster by CheB_2_-P, decreases the sensitivity of the polar cluster over low frequencies.

**Figure 12:**
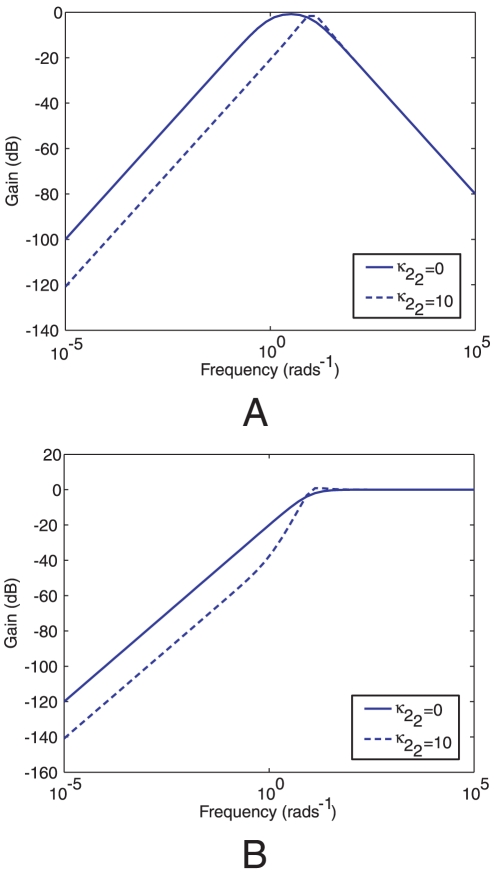
Variation of linear system gain magnitude under different feedback strengths as a function of frequency. Bode magnitude plots of transfer functions from ligand inputs 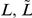 to *Y* in the linear system (1) corresponding to Models I (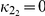) and III (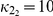). (A) Bode magnitude plots from *L* to *Y*. (B) Bode magnitude plots from 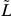 to *Y*.

Figure 12 presents a Bode magnitude plot showing the gain of the linear system (1) to inputs 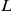 and 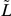 which represent sensed ligand at the polar and cytoplasmic receptor clusters respectively. The figures show that, similar to the simulations of Models I and III, the linear model with a gain 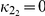 (similar in structure to Model I) and 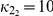 (similar in structure to Model III) also shows a relatively low sensitivity to high frequency (noisy) inputs at the polar receptor cluster and a relatively high sensitivity to noise detected at the cytoplasmic receptor cluster.

## Discussion

From the designed experiments performed, it was possible to invalidate all models but Model III. This suggests that the feedback in the chemotaxis system could occur in an asymmetric fashion. That is, CheB_1_-P may only interact with the membrane signalling cluster whilst CheB_2_-P interacts with both clusters. It is likely that the two chemotaxis pathways initially evolved independently and then became part of the same organism by horizontal gene transfer. Thus one would possibly expect either full connectivity or complete isolation of the two pathways until a further mutation occurs.

### Understanding the outputs of the designed experiments

*R. sphaeroides* has a more complex chemotaxis network than *E. coli* and the multiple receptor clusters and multiple feedback pathways mean that mutants will not always have an intuitive phenotype. For example the Δ*cheB1* mutant does not have the loss of response phenotype one would expect from a direct comparison with the *E. coli* system. We can try to understand why Δ*cheB1* has a steady state at −8 Hz by looking at the structure of the model we have been unable to invalidate, and the reason is as follows: CheB_1_, CheB_2_ and CheY_6_ (along with CheY_3_ and CheY_4_) each compete for phosphoryl groups from CheA_2_-P. CheB_1_ is present in relatively large copy numbers and CheB_1_-P has negligible degradation rate (see Table 1). When present, CheB_1_ ‘stores’ a large proportion of phosphoryl groups. When absent, the competition for phosphoryl groups from CheA_2_-P remains between CheB_2_, CheY_6_, CheY_3_ and CheY_4_. The rate of phosphorylation of CheY_6_ by CheA_2_-P is relatively small, CheY_6_-P receiving most of its phosphorylation from the CheA_3_A_4_-P complex. Therefore deleting *cheB_1_* shifts the equilibrium of the system so that a higher proportion of the phosphoryl groups from CheA_2_-P go to CheY_3_, CheY_4_ or CheB_2_. The increase in CheY_3_-P and CheY_4_-P results in a stronger negative feedback to the cytoplasmic cluster, and the steady-state amount of active receptors at the cytoplasmic cluster is therefore less in the case of Δ*cheB_1_*. The consequence of this is that the main source of phosphorylation for CheY_6_-P, which is CheA_3_A_4_-P, is reduced, and hence the level of CheY_6_-P is reduced. The stopping frequency is consequently reduced. Therefore, rather than Δ*cheB_1_* leading to a loss of response to stimulus, the result of this deletion is a shift in the steady state to a high rotation frequency.

**Table 1:** Model parameters.

Reaction	Parameter(s)	Value(s)
(R_1_) 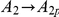	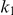	0.03 s^−1^
(R_2_) 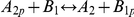	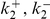	0.035 (µM s)^−1^ , 0.01 (µM s)^−1^
(R_3_) 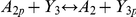	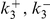	0.065 (µM s)^−1^ , 0
(R_4_) 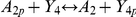	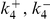	0.004 (µM s)^−1^ , 0
(R_5_) 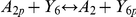	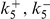	0.0006 (µM s)^−1^ , 0
(R_6_) 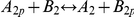	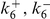	0.0035 (µM s)^−1^ , 0.01(µM s)^−1^
(R_7_) 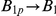	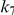	0
(R_8_) 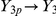	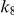	0.08 s^−1^
(R_9_) 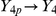	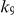	0.02 s^−1^
(R_10_) 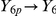	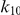	0.1 s^−1^
(R_11_) 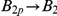	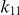	0.015 s^−1^
(R_12_) 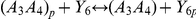	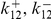	0.1 (µM s)^−1^, 0
(R_13_) 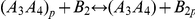	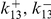	0.006 (µM s)^−1^, 0.07 (µM s)^−1^
(R_14_) 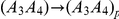	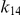	0.02 s^−1^
	CheA 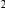	26000 copies per cell
	CheY 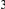	1000 copies per cell
	CheY 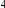	4000 copies per cell
	CheA 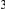 A 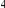	12000 copies per cell
	CheY 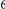	51500 copies per cell
	CheB 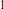	23000 copies per cell
	CheB 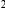	3000 copies per cell

### Relative advantages of the chemotaxis models

The performance measure of Figure 6 suggests that in ascending a ligand gradient under ideal conditions the four models behave almost identically, which may be expected as they all exhibit the same output profile under a step ligand addition. At the same time, simulations of the chemotaxis models showed a difference in robustness between Model III and the other models. From an evolutionary point of view, this may suggest that Model III may have advantages in terms of the robustness of chemotactic performance with respect to the other models. These differences in performance and their implications for chemotaxis are discussed next.

### Sensitivity to parameter variations, noise and ligand inputs

It is desirable that the chemotactic performance of the bacterium is unaffected by changes such as noise in gene expression between the expression of CheOp2 and CheOp3 and therefore the ability to filter out any parametric variations from the pathway's output would be an advantageous feature. The pathway's primary output and the main determinant of chemotaxis performance is the flagellar rotation frequency, which, according to the four models presented, is directly controlled by CheY_6_. It was shown that Models I and III (the latter of which was not invalidated) have a slightly lower sensitivity to variations in the copy number of CheY_6_ compared to Models II and IV (Figure 9). If Model III is indeed valid, such robustness could serve to better maintain the nominal steady state rotation frequency.

**Figure 13:**
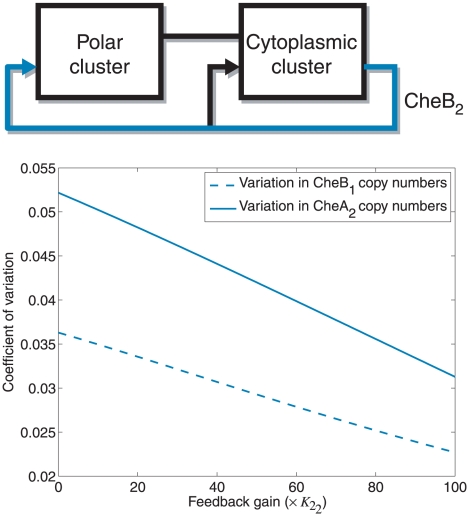
Sensitivity to copy number with varying external feedback. Sensitivity of the chemotaxis steady state to random changes in copy numbers of chemotaxis proteins under different CheB_2_ feedback strengths to the polar cluster. Sensitivity is measured as the ratio of the standard deviation of the steady state to the nominal steady state. Solid line: Sensitivity of the chemotaxis steady state to changes in the copy number of CheA_2_ under different strengths of CheB_2_ feedback to the polar cluster. Dashed line: Sensitivity of the chemotaxis steady state to changes in the copy number of CheB_1_ under different strengths of CheB_2_ feedback to the polar cluster.

Model III also has advantages with respect to Model I due to the CheB_2_-P feedback to the polar cluster. Strengthening this feedback to the polar cluster, which corresponds to increasing the de-methylation rate of the polar cluster by CheB_2_-P, is equivalent to increasing the gain 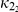 in the linear system (1) – see Figure 10. For the linear model (1), this reduction in sensitivity is illustrated in the Bode sensitivity plot in Figure 11(A). From the point of view of control system design, this feedback is typically used to reduce the magnitude of the system's sensitivity function (see Text S1). This function is dependent on the frequency at which the system is excited and can be shown to be equal to the relative incremental change in the overall system's transfer function in response to an incremental change in the transfer function of the system's sub-modules 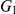 and 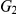. If the sensitivity of the chemotaxis system is low, then the bacterium would be able to maintain its chemotactic response despite changes in the system's biological parameters. The Bode plots (Materials and Methods) in Figure 11(A) illustrate the effect of increasing 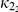 in reducing the sensitivity function of the system (1) over most excitation frequencies. This effect can observed in the chemotaxis models in Figure 9 and Figure 13, where it is shown that strengthening the CheB_2_ feedback to the polar cluster reduces the sensitivity of the steady state rotation frequency to changes in the copy numbers of CheB_1_ and CheA_2_ (see Materials and Methods).

Simulation results in Figure 7 show that the switching frequency in Model III has a low sensitivity to noisy variations in ligand signals detected at the polar receptor cluster relative to the other models. Figure 8 shows the result of a further set of simulations of the four chemotaxis models in which the gain of each chemotaxis model in response to sinusoidal ligand variation detected at the two clusters is given as a function of ligand fluctuation frequency (see Materials and Methods). The figure shows that the switching frequency in Models I and III has a relatively low gain with respect to varying ligand signals detected at the polar receptor cluster and a relatively high gain with respect to ligand variations detected at the cytoplasmic cluster. The Bode magnitude plots in Figure 12 show the frequency-dependent gain of the linear system (1) to sinusoidal ligand inputs in the case 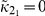, which is structurally similar to Models I and III. These plots parallel the results of the frequency response magnitude plots of Figure 8 which, for Models I and III, show low gain in response to high frequency inputs at the polar receptor cluster and high gain in response to high frequency signals at the cytoplasmic receptor cluster. The rejection of high frequency inputs at the cell pole may be advantageous in that the flagellar switching rate is then only varied when the polar cluster senses a relatively significant ligand concentration gradient that is large in spatial extent, and remains relatively unchanged when the receptors are subject to rapid fluctuations in sensed ligand due, for example, to molecular noise at the receptor such as that simulated in Figure 7.

**Figure 14:**
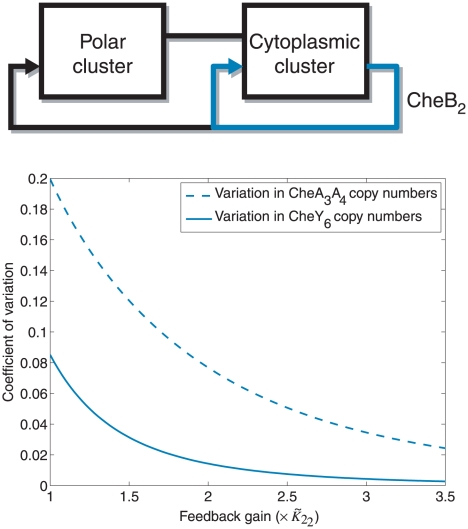
Sensitivity to copy number with varying internal feedback. Sensitivity of the chemotaxis steady state to random changes in copy numbers of chemotaxis proteins under different CheB_2_ feedback strengths to the cytoplasmic cluster. Sensitivity is measured as the ratio of the standard deviation of the steady state to the nominal steady state. Dashed line: Sensitivity of the chemotaxis steady state to changes in the copy number of CheA_3_A_4_ under different strengths of CheB_2_ feedback to the cytoplasmic cluster. Solid line: Sensitivity of the chemotaxis steady state to changes in the copy number of CheY_6_ under different strengths of CheB_2_ feedback to the cytoplasmic cluster.

Although the chemotaxis model assumes that the cytoplasmic cluster input depends on the sensed ligand, it is unknown what the cytoplasmic cluster senses. In addition to the possibility that this input is a function of the sensed ligand concentration, this cluster may potentially also integrate information about the metabolic state of the cell. In this case, this signalling may well be important to chemotactic performance and the relatively high gain of Model III to inputs at the cytoplasmic cluster may suggest that this configuration would favour internal signals over external signals in terms of output. However, if chemotaxis is sensitive to such signals, it would be important that: (i) these signals are tightly controlled and relatively free of the influence of noise and (ii) the cytoplasmic cluster be insensitive to variations in its biological parameters, as sensitivity to such variations would diminish the system's ability to correctly respond to inputs to the cytoplasmic cluster. In Model III, the CheB_2_-P feedback loop around the cytoplasmic cluster could offer this reduction in the sensitivity function of this cluster to such parametric variations. This reduction in sensitivity to variations of cytoplasmic cluster parameters is illustrated in Figure 11(B) using the linear model (1) of the chemotaxis system. The figure shows that increasing the feedback gain 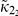, which corresponds to the gain of the CheB_2_-P feedback to the cytoplasmic cluster in Model III, achieves a reduction in the sensitivity of the cytoplasmic cluster. In this way, the cytoplasmic cluster remains sensitive to its inputs, as shown by the large gain at high frequency in Figure 12(B), whilst its sensitivity to parametric variation is reduced due to the internal CheB_2_-P feedback. This effect can be observed in the chemotaxis models in Figure 14, where it is shown that strengthening the CheB_2_ feedback to the cytoplasmic cluster reduces the sensitivity of the steady state rotation frequency to changes in the copy numbers of CheA_3_A_4_ and CheY_6_ (see Materials and Methods).

Figure 8 also shows that for Model I and III, high frequency variations in the ligand concentration sensed at the polar cluster are largely filtered out before causing flagellar switching. This may suggest that the relatively slow dynamics of the polar receptor cluster enable it to function as a low pass filter, preventing any high-frequency noisy variations in the sensed concentration of ligand from being signalled through to the flagellar motor. Figure 12(A) illustrates this attenuation of high frequency polar cluster ligand inputs for the linear model (1).

### Chemotaxis as a cascade controlled system

When combined with the forward signalling pathway which was not invalidated previously [20], Model III has a feedback structure that corresponds to a control scheme termed *cascade control*. This term is used to denote a modular system that includes two feedback loops, one nested within the other. The nested loop is used to regulate a sub-process of the system whilst the ‘external’ negative feedback loop from the system output to the input is used to regulate the entire system.

The measured reaction rates of the two clusters [15], [16] are also such that the cytoplasmic cluster is faster than the polar cluster in responding to inputs, which would be required for the chemotaxis pathway to function as a cascade controlled system [19]. This modularization of the chemotaxis system into fast and slow parts mirrors the division of the cascade controlled system in Figure 1 into the slow and fast subsystems 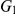 and 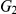 respectively. The cascade control architecture enables the slow (primary) subsystem to fix a set-point for the fast (secondary) system and for the feedback around the secondary system to quickly regulate the secondary output in response to disturbances and variations in the secondary process [19]. This difference in speed is represented by having 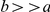, 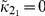 and 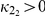 in the linear model (1). Model III also features both an ‘internal’ feedback loop nested within an ‘external’ one corresponding to the dashed and solid feedbacks in Figure 1, respectively. These two feedbacks are manifested by the CheB_2_-P feedback that de-methylates the cytoplasmic and the polar clusters respectively.

Interestingly this architecture mirrors the ability of the system to phosphotransfer, with the membrane cluster being able to phosphotransfer to and be de-methylated by both CheB proteins and the cytoplasmic cluster only phosphotransferring to CheB_2_, the protein that is able to de-methylate it. It does however raise an interesting question. Whereas CheB in *E. coli* is localised to the polar signalling cluster, in *R. sphaeroides* both expressed CheB's are found to be delocalised. Yet, only one of the CheB proteins interacts with both signalling clusters. Thus the advantage of having delocalised CheB_1_ is unclear.

We have shown that if the *R. sphaeroides* chemotaxis pathway has a cascade control architecture, this would enable robust chemotaxis in an uncertain, noisy environment, conferring a selective advantage. In *E. coli*, one feedback loop is used to achieve perfect adaptation and sensing of temporal gradients and because there is only one signalling cluster all signal integration occurs there. Unlike *E. coli*, the *R. sphaeroides* chemotaxis pathway with cascade control feedback provides the bacterium with two feedback loops, one embedded within the other, to adapt and to reduce its sensitivity to parameter variations and noise. The other advantage to this architecture is demonstrated by the simulations shown in Figure 12, which illustrate that with this structure the system would be strongly sensitive to fast-changing inputs to the cytoplasmic cluster, perhaps from the metabolic state of the cell.

Understanding how biological networks achieve robust functionality in the face of disturbances and noise in their internal and external environment is a key question in systems biology. Such networks can be seen as control engineering feedback systems and can be analyzed using system engineering tools in order to understand the advantages of particular internal connectivities over others. In line with this methodology, this paper first utilized a network discrimination approach [20] to construct a model of the feedback connectivity within the *R. sphaeroides* chemotaxis pathway, and then explained the robustness properties of that model by re-interpreting the theoretical advantages of its cascade control structure in a biological framework and comparing it to the other possible models. This suggests a mechanism by which the bacterium can achieve robust chemotactic performance despite biochemical parameter variations and noise. Given that many chemotactic systems have multiple homologues [10] it would appear that using more complex feedback architectures to improve performance may be common in chemotaxis and in other signalling pathways, raising the possibility that this methodology can be used to analyze a wide set of biological systems.

## Materials and Methods

### Modelling the chemotaxis pathway in *R. sphaeroides*

In the next three subsections, we present the three different modules of the chemotaxis signalling pathway: sensing, transduction and actuation.

### Sensing

We assume the same underlying mechanisms for the polar (MCP) and the cytoplasmic (Tlp) receptors. The parameters of the Tlp cluster are labelled with a tilde superscript. We also make the same assumptions of our model as those in [20], which are adopted from the *E. coli* chemotaxis literature [23].

With the notation defined in Table 2, the model for the sensing mechanism is as follows:
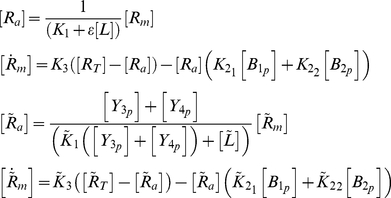
(2)


**Table 2:** Model notation.

Species	Definition
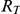	Total polar cluster receptors
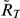	Total cytoplasmic cluster receptors
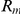	Methylated polar cluster receptors
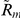	Methylated cytoplasmic cluster receptors
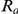	Active polar cluster receptors
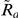	Active cytoplasmic cluster receptors
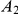	Un-phosphorylated CheA_2_
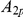	Phosphorylated CheA_2_
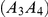	Un-phosphorylated CheA_3_ -CheA_4_
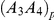	Phosphorylated CheA_3_ -CheA_4_
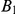	Un-phosphorylated CheB_1_
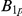	Phosphorylated CheB_1_
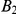	Un-phosphorylated CheB_2_
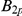	Phosphorylated CheB_2_
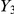	Un-phosphorylated CheY_3_
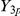	Phosphorylated CheY_3_
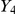	Un-phosphorylated CheY_4_
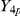	Phosphorylated CheY_4_
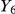	Un-phosphorylated CheY_6_
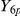	Phosphorylated CheY_6_
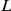	Ligand acting on polar cluster receptors
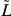	Ligand acting on cytoplasmic cluster receptors
M	Motor activity
*Y*	Average bacterium body rotation rate

We assume that the cytoplasmic receptor cluster senses extracellular ligand concentrations indirectly; for example, 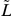 could be internalized attractants, a by-product of the internalization process or a metabolic response to it. For simplicity, we assume the following affine relationship between *L* and 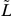


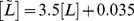
(3)


We let ε = 1 (µM)^−1^ and 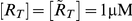. The remaining unknown parameters in this model are the dimensionless quantities 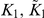, the feedback matrix 
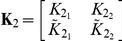
 (which have units of (µM s)^−1^) and 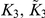 (which have units of s^−1^). The significance of these parameters was detailed in the Results section. We obtain the following values for these unknown parameters for the different models by fitting them to data from tethered cell assays:
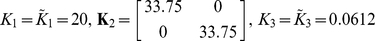


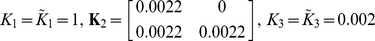


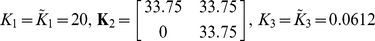


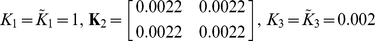



The difference between models I–IV lies in the structure of the CheB_1_-P, CheB_2_-P feedback.

### Transduction

We assume that the structure of the phosphotransfer network is the same as that of the models presented previously in [20], with the modification that when polar and cytoplasmic receptors are in their active state the respective auto-phosphorylation rates of CheA_2_ and CheA_3_, 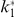 and 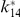, are accelerated to 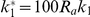 and 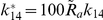 where 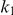 and 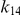 are the reaction constants of the auto-phosphorylation of CheA_2_ and CheA_3_ obtained from *in vitro* experiments in the absence of the influence of receptors, as given in Table 1 and in [20]. Biologically, it would be expected that the auto-phosphorylation rates 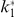 and 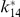 (for the case where CheA_2_ and CheA_3_ are each in a fully active complex) are higher than the rates 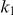 and 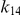 measured *in vitro*.

### Actuation

We denote the flagellar stopping frequency by *M*. We assume some interaction which does not lead to a long lasting binding between CheY_6_-P and the FliM rotor switch. However, stopping frequency decreases at a constant rate in the absence of CheY_6_-P. This relationship between the CheY_6_-P and the stopping frequency effectively constitutes a low-pass filter that attenuates fast changes in CheY_6_ -P concentration. We model this behaviour by:
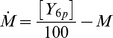
(4)


The output of the model is the flagellar rotation frequency observed in tethered cell assays. We use the following heuristic description to convert motor activity into *R. sphaeroides* body rotations (given in rot/sec or Hertz):
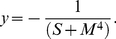
(5)


We set 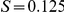 which means that saturation occurs at −8 rot/sec. This value follows from experimental observations – even for major changes in attractant concentrations this value was almost never surpassed.

## Measuring chemotactic performance

The measure of chemotactic performance used in the paper is the relative distance travelled by the bacterial cells up an attractant gradient. The medium in which the cells chemotax is assumed to be a two-dimensional plane having an *x*- and a *y*- dimension (where distance along these two directions is unit-less), as illustrated in Figure 6 (left). The ligand concentration *L* is assumed to vary as *L* = 100*x* (for *x*>0) and *L* = 0 otherwise, remaining unchanged along the *y* direction. The simulation is initialized with the bacterial cells having a starting position of *x* = 0 and an initial orientation aligned with the positive *x* direction.

At each switch, the bacterium is assumed to change its orientation by an angle (measured in radians) randomly selected from the zero-mean, unity-variance Gaussian distribution. The concentration of ligand at its position, is then input into the chemotaxis model described above. The output, the flagellar rotation frequency (in Hz), is then translated to the size of the step the bacterium makes in the direction of its orientation.

## Parametric sensitivity analysis

To measure the effect of the variation of a particular parameter on the steady state flagellar rotation frequency, several values of the parameter of interest were randomly selected from a normal distribution with a mean given by the nominal value of the parameter for the given model and with a standard deviation given by half the nominal value of the parameter. A simulation of the model at steady state was then run and the resulting steady state rotation frequency was recorded for each of the randomly chosen parameter values. The coefficient of variation, given by the ratio of the standard deviation of the recorded steady state values to the nominal steady state value was then computed. This dimensionless quantity can be used to compare the dispersion of quantities with a non-zero mean. Sensitivity to a certain parameter value is therefore high when its corresponding coefficient of variation is high, as this would indicate a significant shift from the nominal output in response to a variation in parameter values.

## Linear systems analysis techniques

To compare the different chemotaxis feedback structures in an analytical way, the linear system (1) was constructed. A rich theory exists to analyze and compare the properties of linear systems in the so-called frequency domain using their associated transfer functions [25]. Using such tools, it is possible to study the effects of excitation frequency on systems' gains and sensitivities as was done in this paper. As an example of how this method works, consider a linear dynamical system
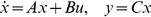
(6)where *A*, *B* and *C* are matrices of appropriate dimension, whose entries depend on the model parameters, and 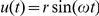 is a sinusoidal input with angular frequency 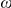 and fixed amplitude *r*. System (6) is the so called state space representation of the model in the time domain. It is common in control systems engineering to investigate the behaviour of such a system's dependency on excitation frequency 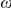. This requires transforming the system to the frequency domain via the Laplace transform. We denote the Laplace transform of *u* and *y* by *U*(*s*) and *Y*(*s*) respectively, where *s* is a complex independent variable. Then,
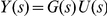
where *G*(*s*) is the transfer function in the frequency domain and is given by [25]:
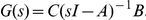



By evaluating this function for values of *s* on the imaginary axis (by setting 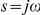 where *j* is 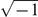) we obtain a frequency domain relationship between the system's input and output. If the system is stable (the eigenvalues of matrix *A* have negative real parts) and is excited with a periodic input signal *u* of frequency 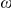, then after some transient behaviour the output *y* is given by a sinusoidal wave that is phase shifted and amplified with respect to *u* by amounts dependent on 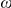. The amplification factor is given in decibels by
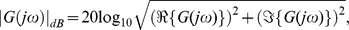
whilst the phase shift is given by
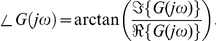



The Bode magnitude plot shows the variation of 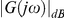 in decibels with frequency of excitation 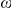. The Bode phase plot shows the variation of 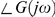 in radians with frequency of excitation 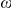.

## Model parameters

Model parameters were obtained by performing least squares fitting on previously obtained experimental data [15], as described in [20]. These are listed in Table 1. Protein concentrations were obtained via quantitative western blotting as described in [20].

## Response to noisy ligand input

We tested the gain of each model to sinusoidally varying ligand input signals, applied separately at the polar and at the cytoplasmic clusters. In the first case we applied the constant ligand input 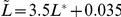, with 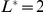 to the cytoplasmic cluster whilst simultaneously applying to the polar cluster sinusoidally varying ligand signals given by 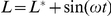, with frequency 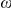 in the range 0.01 to 1 rads^−1^.

In the second case we applied the constant ligand input 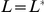 to the polar cluster whilst simultaneously applying to the cytoplasmic cluster sinusoidally varying ligand signals given by 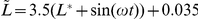, with frequency 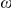 in the range 0.01 to 1 rads^−1^.

The frequency response magnitude plots of Figure 8 show the magnitude of the fundamental frequency of the sinusoidal variation in the flagellar rotation frequency in response to these sinusoidal ligand input signals.

## Plasmids and strains

The strains used in this study are shown in Table 3. *R. sphaeroides* strains were grown in succinate medium at 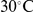 under aerobic conditions with shaking. Where required, nalidixic acid was used at concentrations of 25 g ml^−1^.

**Table 3:** Strains used in this study.

Strain/Plasmid	Characteristics	Source
*R. sphaeroides* WS8N	Spontaneous nalidixic acid resistant mutant of wild type WS8	[26]
*R. sphaeroides* JPA517	WS8N with the *cheB_1_* gene deleted by genomic replacement	[17]
*R. sphaeroides* JPA 1320	WS8N with the *cheR_3_* gene deleted by genomic replacement	[15]

## Tethered cell analysis

Tethered cell responses to propionate of the *R. sphaeroides* strains were characterized as described previously [20]. For each strain and wild type 4 slides were analyzed each containing 10 cells.

## Supporting Information

Text S1 Supporting information text.
(0.95 MB DOC)
