# Supplementary material for: Feedback Control Architecture and the Bacterial Chemotaxis Network
Source: PLoS Comput Biol. 2011 May 5;7(5):e1001130. doi: 10.1371/journal.pcbi.1001130 (PMC3088647; doi:10.1371/journal.pcbi.1001130)
Supplement: Text S1 — Supporting information text. (0.95 MB DOC) [file pcbi.1001130.s001.doc]

**Text S1**

**Feedback Control**

The primary aim of using feedback in a control system is to reduce the effect of uncertainties in the system and thus maintain the desired output. Consider the block diagram in Figure S1 which models a plant *P*(*s*) in feedback with a controller *F*(*s*). The closed loop transfer function from input *u*(*t*) to output *y*(*t*) is given by

| $Y(s)=\frac{P(s)}{1+F(s)P(s)}U(s)=T(s)U(s)$ |  |
| --- | --- |

where *U*(*s*) and *Y*(*s*) are the Laplace transforms of *u*(*t*) and *y*(*t*) respectively. The objective here is to obtain a desired output from plant *P*(*s*). The plant is typically subject to uncertainties and un-modelled plant dynamics, which will ultimately affect the input-output transfer function *T*(*s*). The effect of an incremental change in the plant *P*(*s*) on transfer function *T*(*s*) can be quantified by taking the normalized derivative of a change in *T*(*s*) with respect to a change in *P*(*s*):

| $\frac{\frac{\partial T(s)}{T(s)}}{\frac{\partial P(s)}{P(s)}}=\frac{1}{1+F(s)P(s)}=S(s)$ |  |
| --- | --- |

where *S*(*s*) is called the Sensitivity function. The feedback controller *F*(*s*) can be used to reduce the sensitivity of *T*(*s*) to changes in the plant *P*(*s*) over a range of frequencies.

Cascade Control

|  | (1) |
| --- | --- |

In this section we discuss how a cascade control scheme can achieve improved system performance. As a simple example demonstrating cascade control, we consider the system described in the frequency domain by

which is schematically illustrated in Figure S2. This system is the linear representation of Model III of the chemotaxis pathway of *R. sphaeroides* given in (1) and perturbed with the additional disturbance signal *d*(*s*). We assume that $a,\text{ }b,\text{ }\kappa_{2_{2}},\text{ }\tilde{\kappa}_{2_{2}}>0$ and that $b>a$, which means that the isolated cytoplasmic cluster responds more quickly to signals than the polar cluster and has a higher cut-off frequency. A cascade control architecture involves placing an ‘internal’ negative feedback of gain $\tilde{\kappa}_{2_{2}}$around the faster module of the system, shown as the dashed-dotted line in Figure S2 in addition to an output feedback of gain$\kappa_{2_{2}}$. Integral control guarantees that when $d=0$ the levels of signals CheB_1_-P and CheB_2_-P in Figure S2 are restored to zero in the steady state, regardless of the values of $a,b,\kappa_{2_{2}}$and $\tilde{\kappa}_{2_{2}}$.

The cascade control scheme results in a higher bandwidth, increased damping and better rejection of disturbances *d* compared to a simple feedback architecture. In addition, high values of $b,\kappa_{2_{2}}$and $\tilde{\kappa}_{2_{2}}$ cause the feedback to reduce the magnitude of the sensitivity function of the system to parametric variations and uncertainties. The following subsections will discuss the ways in which cascade control achieves these improvements in system performance. First, note that the ‘poles’ of the system, i.e. the values for which the denominator of the right hand side of (1) is zero, satisfy

| $(s+a)(s+b\tilde{\kappa}_{2_{2}})+ab\kappa_{2_{2}}$=0.  From this characteristic equation, we arrive at the following properties. |  |
| --- | --- |

Higher bandwidth

Comparing the above equation with the standard characteristic equation of a second-order system,

| $s^{2}+2\zeta\omega_{n}s+\omega_{n}^{2}=0$ |  |
| --- | --- |

where $\omega_{n}$is the undamped natural frequency of the system and$\zeta$is the damping factor, gives a natural frequency of $\omega_{n}=\sqrt{ab(\kappa_{2_{2}}+\tilde{\kappa}_{2_{2}})}$ for the system, which increases with $a,b,\kappa_{2_{2}}$and $\tilde{\kappa}_{2_{2}}$. Hence cascade control (having a high $\tilde{\kappa}_{2_{2}}$) increases the bandwidth of the system reducing its settling time and improving its set-point tracking.

Increased damping

Following from the above, the damping ratio $\zeta$ of this system is given by $\zeta=\frac{a+b\tilde{\kappa}_{2_{2}}}{2\sqrt{ab(\kappa_{2_{2}}+\tilde{\kappa}_{2_{2}})}}$. It can easily be shown that for $\kappa_{2_{2}}>0$,$\tilde{\kappa}_{2_{2}}>0$ the damping is an increasing function of $\tilde{\kappa}_{2_{2}}$if $b>\frac{a}{2\kappa_{2_{2}}+\tilde{\kappa}_{2_{2}}}$.

Disturbance rejection

From (1), the transfer function from the disturbance signal *d* to the output *y* is given by $\frac{b(s+a)}{(s+a)(s+b\tilde{\kappa}_{2_{2}})+ab\kappa_{2_{2}}}$. Using the Final Value Theorem, the steady-state response to a step increase in the disturbance *d* of magnitude $\hat{d}$ will be a constant signal of magnitude. Therefore strengthening the cascade control feedback gain reduces the steady-state effect of the disturbance *d* on the output *y*.

Sensitivity

The cascade control feedback configuration reduces the magnitude of the sensitivity functions of both the overall system and the cytoplasmic cluster. The two sensitivity functions can be derived as above. For the isolated cytoplasmic cluster the sensitivity $S_{cyt}$is

| $S_{cyt}=\frac{s}{s+b\tilde{\kappa}_{2_{2}}}$. |  |
| --- | --- |

The magnitude of this sensitivity function is reduced by having a large feedback gain$\tilde{\kappa}_{2_{2}}$. Similarly, the sensitivity of the transfer function from $\tilde{L}$to *Y* to variations in the transfer function from $\tilde{L}$to the signal CheB_2_-P in Figure S2 is given by

| . |  |
| --- | --- |

As shown in the paper, a high value of $\kappa_{2_{2}}$ reduces the magnitude of $S_{\tilde{L}\to Y}$over most frequencies.

In the case of Model III of the *R. sphaeroides* chemotaxis pathway given in the paper, it was shown in Figure 8 that there is a relatively high gain from signals input at the cytoplasmic cluster to the flagellar output of the model. If Model III is accurate, this would suggest that signals input to the cytoplasmic cluster have a significant effect on the chemotaxis pathway’s output and therefore need to be tightly controlled. Furthermore, the output of the cytoplasmic cluster needs to be insensitive to parametric variations in order to accurately convey signals sensed at its inputs to the flagellar motors. The CheB_2_-P feedback that demethylates active cytoplasmic cluster receptors has an analogous role to the$\tilde{\kappa}_{2_{2}}$feedback above, reducing the magnitude of the sensitivity function of the cytoplasmic cluster. In this way, uncertainties and parametric changes have a reduced effect on the system’s flagellar output.

Example - High performance aircraft pitch autopilot

To illustrate an application of cascade control we shall look at an example of pitch control for a high performance aircraft, adapted from []. Figure S3 is a block diagram of the jet aircraft’s short-period longitudinal behaviour. Output *Y* is the aircraft’s pitch and *R* is a reference signal. With$S_{r}=0$, the response of the system is very oscillatory, and its damping ratio decreases further when $S_{a}$ is increased. Moreover, a relatively small increase in the gain$S_{a}$of 3.84 is enough to cause instability. The damping and gain margin can be increased through placing a feedback loop of gain$S_{r}$ as illustrated by the dashed line in Figure S3. This can be accomplished using a rate gyro. By setting $S_{r}=1.98$the damping ratio is increased and the system will remain stable for a gain $S_{a}$ of up to 24.6. For further details, see [].

Example - Heat Exchanger

To further demonstrate the disturbance rejection properties of cascade control, we shall consider a heat exchanger example. The role of the heat exchanger is to transfer heat from steam to an effluent. The temperature set-point of the effluent is fixed in the temperature controller module of Figure S4 and the aim of the control system is to maintain the effluent temperature at this set point.

Without cascade control, the temperature controller measures the effluent temperature and sets a reference flow rate for the flow controller to follow. The flow controller then controls the opening of a valve that sets the flow rate. However, upstream pressure disturbances may cause the steam flow rate to change abruptly. This disturbance will only be corrected for once the change in steam flow affects the temperature of effluent, which may be a slow process.

Cascade control involves taking a measurement of the steam flow rate and feeding this measurement into the flow controller (shown in the dashed line in Figure S4). If the flow rate is then found to have deviated from the reference flow rate set by the temperature controller, the flow controller itself corrects the flow rate by opening or closing the flow valve as necessary. This corrective measure is a more rapid way of maintaining the required flow rate than via the temperature feedback alone.

Block diagram representations of this system (including set-point *r* and disturbance signals *d*) with and without cascade control are shown in Figure S5 and Figure S6. The parameter values are $a=0.2,\text{ }b=30,\text{ }k=10.$Note that the flow controller, around which we have the cascade control feedback, is much faster than the temperature controller. Figure S7 illustrates the response to a step change in the set-point temperature $r=10$ (at 50 seconds) and a step output disturbance $d=10$ (at 250 seconds) for the system with and without cascade control.

Cascade control therefore results in a less oscillatory response and one that more rapidly rejects disturbances than a system without this control scheme.

**References**

[1] Blakelock, JH (1991), Automatic Control of Aircraft and Missiles, 2nd edition. Wiley- Interscience, pp. 62-111.

Figure S1: Simple feedback system for plant *P*(*s*) and feedback controller *F*(*s*).

Figure S2: Linear system representation of Model III with a cascade control feedback (dashed-dotted line). Ligand inputs to the two signalling clusters are *L* and $\tilde{L}$ and signal *d* is a disturbance signal. The parameters $a,b,\kappa_{2_{2}},\tilde{\kappa}_{2_{2}}$are positive with$b>a$.

Figure S3: Block diagram of aircraft pitch dynamics. Cascade control manifests in the dashed line. Laplace domain signals *R*(*s*) and *Y*(*s*) are the reference input and system output respectively. $S_{a}$and$S_{r}$are constants and $\frac{1}{s}$ denotes an integrator in the Laplace domain.

*s*

Figure S4: Schematic of a heat exchanger. Cascade control involves feeding a measurement of the flow rate into the flow controller (dashed line).

Figure S5: Heat exchanger system with cascade control: dashed feedback of gain *k* gives improved performance. Signals *r* and *y* are the reference input and system output respectively. Signal *d* is an output disturbance. Parameters *a*, *b*, *k* denote constant gains.

Figure S6: Heat exchanger system without cascade control. Signals *r* and *y* are the reference input and system output respectively. Signal *d* is an output disturbance. Parameters *a* and *b* denote constant gains.

Figure S7: Heat exchanger response to set-point change and disturbance, with cascade control (solid line) and without (dashed line).
